# Toward Uncertainty‐Aware Hemolysis Modeling: A Universal Approach to Address Experimental Variance

**DOI:** 10.1002/cnm.70040

**Published:** 2025-05-14

**Authors:** Christopher Blum, Ulrich Steinseifer, Michael Neidlin

**Affiliations:** ^1^ Department of Cardiovascular Engineering Institute of Applied Medical Engineering, Medical Faculty, RWTH Aachen University Aachen Germany

**Keywords:** hemolysis modeling, in silico, Markov chain Monte Carlo, uncertainty quantification hemolysis

## Abstract

The purpose of this study is to address the lack of uncertainty quantification in numerical hemolysis models, which are critical for medical device evaluations. Specifically, we aim to develop a probabilistic hemolysis model, which incorporates experimental variability using the Markov Chain Monte Carlo (MCMC) method to enhance predictive accuracy and robustness. Initially, we examined the objective function landscape for fitting a Power Law hemolysis model, whose parameters are derived from inherently uncertain experimental data, by employing a grid search approach. Building on this, we applied MCMC to derive detailed stochastic distributions for the hemolysis Power Law model parameters *C*, *α*, and *β*. These distributions were then propagated through a reduced order model of the FDA benchmark pump to quantify the experimental uncertainty in hemolysis measurements with respect to the predicted pump hemolysis. Our analysis revealed a global flat minimum in the objective function landscape of the multi‐parameter power law model, a phenomenon attributable to fundamental mathematical limitations in the fitting process. The probabilistic hemolysis model converged to a constant optimal *C* = 3.515 × 10^−5^ and log normal distributions of *α* and *β* with means of 0.614 and 1.795, respectively. This probabilistic approach successfully captured both the mean and variance observed in the experimental FDA benchmark pump data. In comparison, conventional deterministic models are not able to describe experimental variation. Incorporating uncertainty quantification through MCMC enhances the robustness and predictive accuracy of hemolysis models. This method allows for better comparison of simulated hemolysis outcomes with in vitro experiments and can integrate additional datasets, potentially setting a new standard in hemolysis modeling.

## 1 Introduction

Hemolysis, the destruction of red blood cells, presents a significant challenge in the development and use of cardiovascular blood‐contacting devices. When red blood cells are damaged, hemoglobin is released into the bloodstream, potentially causing serious complications. This phenomenon is particularly relevant in devices such as mechanical heart valves [1], ventricular assist devices (VADs) [2], and other forms of mechanical circulatory support (MCS) [3, 4]. These life‐saving devices can exert mechanical forces that rupture red blood cells, leading to hemolysis. Due to supraphysiological shear rates, especially in MCS devices, this is a major concern as high levels of hemolysis are associated with increased mortality [5], thrombosis, and bleeding events [2, 6]. Therefore, mitigating hemolysis is crucial for improving the safety and efficacy of these devices.

To address this issue, researchers employ in silico numerical hemolysis models to optimize MCS designs, aiming to reduce adverse events. In silico models are cost‐effective alternatives to in vitro and in vivo studies, saving both time and resources that are applied across various disciplines [7, 8, 9]. Recognizing the importance of in silico models, the FDA has recently issued guidelines supporting the use of computational models for device approvals, emphasizing the need for reliable and accurate computational models [10]. Central to these guidelines are verification, validation, and uncertainty quantification (VVUQ), which ensure the reliability of the computational model and account for real‐world data variations.

Given the emphasis on uncertainty quantification (UQ), it is essential to examine the current computational gold standard for predicting hemolysis: the Power Law method introduced by Giersiepen et al. [11]. This method is based on experimental measurements that describe the relationship between hemolysis, shear stress, and exposure time. It is generally known that with increased shear stress and or exposure time an increase in hemolysis is seen. To use this relationship in computational models, the experimental data is fitted using a power law approach $\mathrm{HI}=C*\tau^{\beta }*t^{\alpha }$ providing a functional relationship between these variables. Despite its utility, the Power Law method primarily allows for relative comparisons rather than predicting absolute hemolysis values. Numerous studies have attempted to refine this approach by investigating blood from different species [11, 12, 13] employing various apparatuses [13, 14, 15, 16] or employing different numerical techniques [17, 18, 19, 20]. Recent works have used particle optimization algorithms for parameter identification [21] or have focused on turbulent energy dissipation as a marker for hemolysis [22]. However, no universal parameter set for the Power Law method has been established yet. A detailed review of hemolysis prediction with CFD models is presented by Yu et al. [23] and a more general overview of shear‐induced hemolysis has been performed by Faghih et al. [24].

A critical limitation of Power Law models is their inability to account for inherent variance in experimental data, as the fitting process results in a loss of this variance, leading to a representation that only captures an average scenario of hemolysis. This raises concerns about the robustness of the fitting approach used to determine the optimal model parameters (*C*, *α*, and *β*), as it should accurately mirror the original hemolysis signal of the underlying experiment. Notable attempts to address this robustness include Mohammadi et al.'s [25] adjustment of model parameters to calculate probability distributions of the model parameters and Craven et al.'s [26] device‐specific hemolysis model, that relied on a combination of computational models and experimental device hemolysis data. Both studies highlighted a notable sensitivity of resulting hemolysis to changes in model parameters, underlining the importance of finding the correct parameters. However, both approaches lacked consideration of experimental variance in their models.

To overcome these limitations and meet the increased importance of reliable and robust hemolysis models incorporating UQ, this study aims to develop a universal method for analyzing all experimental hemolysis data sets, incorporating the variance of underlying experiments. By accounting for experimental variance, we can enhance the reliability and robustness of hemolysis predictions, ensuring that future regulatory submissions for blood‐contacting medical devices are based on more robust and accurate models.

## 2 Materials and Methods

**FIGURE 1:**
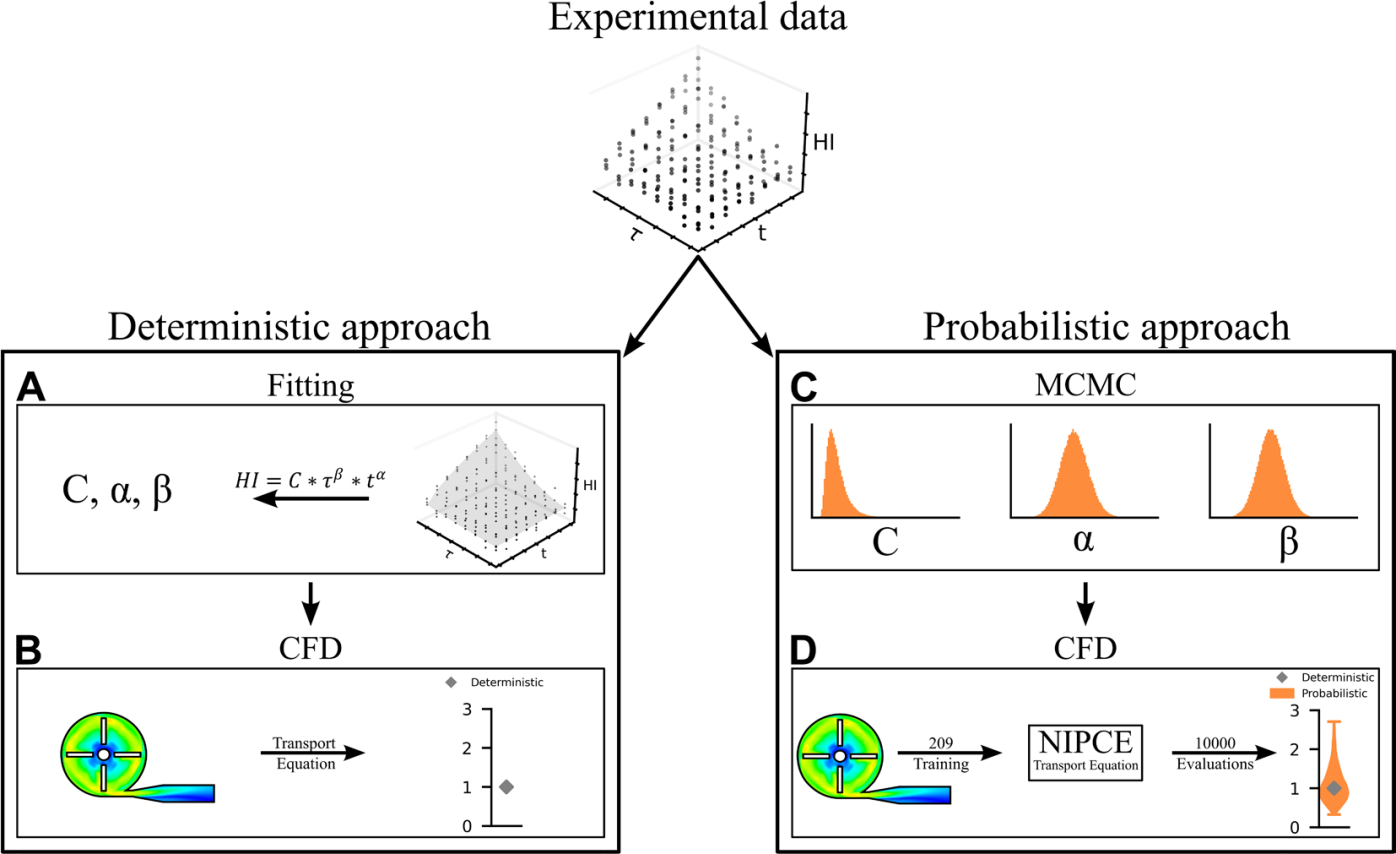
Graphical overview of deterministic and novel probabilistic approach. Starting from experimental data, (A) and (B) show the traditional deterministic approach with experimental data fitting and CFD solution steps, respectively. In (C) and (D), the probabilistic approach is detailed with MCMC and CFD solution, including NIPCE calculation, respectively.

Figure 1 provides a visual summary of the analyzes conducted and methods employed in this study. The study uses experimental data describing the relationship between Hemolysis Index (HI), exposure time (*t*), and shear stress (τ) with a repetition number of *n* = 3 at each operating point, utilizing a custom‐built Couette‐type shearing device. The exposure time ranged from 0.039 s to 1.48 s, and the shear stress ranged from 50 to 320 Pa, using ovine blood as the test species [27]. It compares the deterministic approach to determining hemolysis in a device with a novel probabilistic approach. At first the deterministic approach is analyzed in detail, revealing the relationship between experimental observations and model predictions. Then the probabilistic approach including the Bayesian parameter estimation method Markov Chain Monte Carlo (MCMC) and the reduced order model technique non‐intrusive polynomial chaos expansion (NIPCE) is shown. In the end, the deterministic and probabilistic hemolysis models are compared using data from the FDA benchmark pump [28].

For easy reproducibility of the presented probabilistic approach, a python script to create the MCMC model as well as synthetic data to test the model can be found at https://zenodo.org/records/12820264.

### 2.1 Deterministic Approach

Using Equation (1), the deterministic approach begins with fitting a Power Law model to the experimental data describing the relationship between shear stress, exposure time, and hemolysis under well‐controlled experimental conditions. For this, various fitting algorithms can be employed to achieve an optimal solution based on all experimental data, described by the optimal parameters *C*, *α*, and *β* of Equation (1).

(1) $$\mathrm{HI}=C*\tau^{\beta }*t^{\alpha }$$

Equation (1), with the optimal fitted parameters *C*, *α*, and *β*, can be utilized to predict deterministic hemolysis values (e.g., MIH) in more complex flow scenarios typically encountered in realistic blood‐carrying devices. To achieve this, Equation (1) is reformulated into a transport equation and solved using Computational Fluid Dynamics (CFD) under specified boundary conditions.

For all results presented using the deterministic approach, the original model parameters determined by the multivariate regression method from Zhang et al. [27] were used (*C* = 1.228 × 10^−5^, *α* = 0.6606, *β* = 1.9918).

Regardless of the fitting method, the sum of squared errors (SSE) between the experimental observations and model predictions can be calculated:

(2) $$\mathrm{SSE}=\sum_{i=1}^{n}{\left(y_{i}-{\hat{y}}_{i}\right)}^{2}$$

Here, $y_{i}$ represents the experimental observation of HI, and ${\hat{y}}_{i}$ denotes the model prediction of Equation (1) with specific Parameters *C*, *α*, and *β*. The variable n describes the number of observed experimental values. To explore the possible parameter space and identify potential multiple optimal solutions of the deterministic approach from Equation (1), an initial global grid search with broader parameter ranges was conducted to determine appropriate ranges for a more detailed analysis. Subsequently, a refined grid search was performed over the narrowed values for *C* (1 × 10^−5^ to 1 × 10^−3^), α (0.1–1), and *β* (1.2–2.5), with 1000 values for *α* and *β* and 12 for *C*, resulting in 12,000,000 possible combinations that were analyzed.

### 2.2 Probabilistic Approach

The probabilistic approach uses the same experimental data as the deterministic approach, employing the MCMC method to determine the posterior distributions of the model parameters in Equation (1) with the highest likelihood of observing the provided experimental data. This is in contrast to the deterministic approach, which minimizes one objective function and delivers only one fitting parameter set.

The samples drawn from the posterior distributions of *C*, *α*, and *β* are transformed into hemolysis values using CFD, similar to the traditional approach. To achieve a meaningful posterior distribution of hemolysis values, this sampling process must be conducted very frequently. A reduced‐order model of the CFD simulation, created using NIPCE, substantially accelerates the prediction of hemolysis based on the model parameters.

### 2.3 MCMC Implementation

MCMC methods in Bayesian statistics estimate posterior distributions of model parameters by iteratively updating prior assumptions based on observed data. The prior distribution represents the initial beliefs about the possible values of the parameters before incorporating experimental data. The posterior distribution, in contrast, is the updated probability distribution of the parameters after integrating the experimental evidence using Bayes' theorem. Through repeated sampling, MCMC refines this distribution, allowing an unbiased exploration of the parameter space while accounting for uncertainties in both the prior knowledge and the observed data. Further introductory details of this method can be found in [29, 30]. By leveraging MCMC, this study accounts for uncertainties in experimental hemolysis data, refining the parameter fitting process and improving the reliability of numerical hemolysis predictions. This study used the Python package PyMC [31] to implement MCMC, with experimental data from Zhang et al. [27] and Equation (1) serving as the basis for sampling posterior distributions. An additional parameter *σ* was introduced to capture the variability in the experimental data that cannot be explained by the model parameters *C*, *α*, and *β*. To avoid introducing excessive domain knowledge into the prior assumptions, we opted for uninformed priors. Specifically, the prior distributions were set as uniform (0–1) for C and normal (mean = 0, std. = 1) for the other parameters. While defining normal distributions around deterministic values could potentially improve MCMC convergence, we chose this uninformed approach to ensure that the posterior distributions were determined solely by the experimental data. This decision prevents prior assumptions from influencing the results and ensures an unbiased exploration of parameter uncertainties.

The likelihood of observing the hemolysis index (HI) was modeled by a Student's t‐distribution:

(3) $$\mathrm{HI}\sim t\left(\mu ={\hat{y}}_{i},\sigma =\sigma ,\nu =\frac{1}{30}\right)$$

where μ represents the central value around which the data is distributed. For each combination of *C*, *α*, and *β* this central value is given by Equation (1). The scale parameter *σ* describes the spread of the data around the central value, capturing the measurement noise variability during the sampling process. The heaviness of the tails (degrees of freedom) is described by, following an exponential distribution with a rate parameter of 1/30. The Student's t‐distribution was chosen for its robustness to outliers, effectiveness with small sample sizes, and ability to handle uncertainty in variance estimates. During MCMC sampling, *σ* is sampled alongside *C*, *α*, and *β*. The sampler explores the joint posterior distribution of all parameters, accounting for the interdependencies between them. This simultaneous sampling ensures that the uncertainty in *σ* is properly propagated to the estimates of *C*, *α* and *β*. This means that *σ* directly affects the probability of observing the data given the model parameter,s and that the posterior distributions of *C*, *α* and *β* incorporate the uncertainty in *σ*. Supporting Information on this statement can be found in the Supporting Information Figure S2.

Using the No‐U‐Turn Sampler (NUTS) algorithm [32], four chains with 50,000 samples each were sampled after a burn‐in period of 1000 samples and with a target acceptance rate of 0.95. The resulting 200,000 samples per MCMC run provided the basis for the posterior distributions. Chain convergence was assessed using trace plots and the Gelman‐Rubin Diagnostic, with an example trace plot provided in the Supporting Information (Figure S1).

Models with a constant *C* parameter were also computed. For different values of *C*, the distributions of *α*, *β*, and *σ* took on different forms. Bayesian optimization identified the *C* value that yielded the smallest median value of the *σ* distribution. For this, *C* was varied within the bounds of 1 × 10^−6^ to 1 × 10^−3^, and the optimal value of *C* = 3.515e‐5 was found within 100 iterations using the *gp_minimize* function of the Python package *scikit‐optimize* [33] (Figure S5).

### 2.4 CFD Model

To compare the probabilistic and deterministic methods, the study utilized the FDA Round Robin benchmark blood pump [28]. This blood pump has been extensively investigated for hemolysis in a multicenter study [34, 35], making it an ideal basis for comparing hemolysis predictions from the models with experimental and simulation data. The validation of the Computational Fluid Dynamics (CFD) model was previously conducted by our research group, as detailed by Gross‐Hardt et al. [36]. For comprehensive information regarding the mesh and simulation parameters, please refer to their publication.

To summarize, the geometry was meshed using the ANSYS 2021 R1 Meshing tool (ANSYS Inc., Canonsburg, USA), employing unstructured tetrahedral elements with prism layers. This meshing approach resulted in a total element count of 9.3 million cells, corresponding to the “medium” mesh configuration described in the earlier study [36]. The CFD simulations were performed using the ANSYS CFX solver, with the k‐ω shear stress transport model selected for turbulence modeling. Unlike Gross‐Hardt et al. [36], steady‐state simulations were conducted to reduce computational effort for generating training data. This approach was validated against experimental data [34], demonstrating that the simplification to steady‐state simulations is justified due to the minimal differences observed when compared to unsteady simulations and the validation dataset. For hemolysis calculations, the shear stress tensor was reduced to a scalar shear stress (SSS) for cartesian coordinates *x*, *y*, *z*, velocity components *u*, *v*, *w*, and the dynamic viscosity η using Equation (4).

(4) $$\mathrm{SSS}=\eta {\left[2\left\{{\left(\frac{\partial u}{\partial x}\right)}^{2}+{\left(\frac{\partial v}{\partial y}\right)}^{2}+{\left(\frac{\partial w}{\partial z}\right)}^{2}\right\}+{\left(\frac{\partial u}{\partial y}+\frac{\partial v}{\partial x}\right)}^{2}+{\left(\frac{\partial u}{\partial z}+\frac{\partial w}{\partial x}\right)}^{2}+{\left(\frac{\partial v}{\partial z}+\frac{\partial w}{\partial y}\right)}^{2}\right]}^{\frac{1}{2}}$$

With this scalar shear stress, the hemolysis value for each simulation was calculated in a post‐processing step using the volume integral formulation (Equation (5)) of Garon and Farinas [37] resulting in a numerical equivalent of the modified index of hemolysis (MIH).

(5) $$\mathrm{MIH}={\left(\frac{1}{Q}\int_{V}{\left(C\tau^{\beta }\right)}^{\frac{1}{\alpha }}\mathrm{dV}\right)}^{\alpha }10^{6}$$

With *Q* being the blood volume flow rate of the inlet and *V* the volume of the whole computational domain. With this formulation, Gauss's theorem ensures that, in a steady‐state flow and assuming zero HI at the inlet, the volume integral of the transported HI species over the entire computational domain is equivalent to its surface integral over the outlet. As a result, once the flow field has been computed, hemolysis can be evaluated for different sets of fitting parameters through a simple post‐processing step by calculating the relevant volume integral. This approach streamlines the analysis, as it avoids the need to rerun the full CFD simulation for each parameter combination.

### 2.5 Reduced‐Order Model

Reduced‐order modeling (ROM) is a computational technique used to approximate computationally intensive simulations with substantially lower computational cost while maintaining accuracy. In the context of this study, we apply Non‐Intrusive Polynomial Chaos Expansion (NIPCE), a surrogate modeling approach that efficiently calculates hemolysis values for 10,000 variations of fitting parameters. NIPCE works by approximating the relationship between input parameters (fitting parameters) and output responses (MIH) using an expansion of orthogonal polynomials. Instead of solving Equation (5) for each new fitting parameter set, NIPCE expresses the output quantity (MIH) as a weighted sum of polynomial basis functions of the input parameters. The coefficients of this polynomial expansion are determined through regression, using a set of training data generated from evaluations of Equation (5). Once trained, the NIPCE model provides rapid evaluations of new parameter sets by simply evaluating the polynomial approximation rather than running the more computationally expensive task of solving Equation (5). The MCMC posterior distributions were fitted using log‐normal distributions truncated at the lower 1% and upper 99% intervals to avoid extreme values in the subsequent sampling process (Figure S3). These distributions were then combined into a multivariate distribution. Next, 200 samples were drawn from this multivariate distribution using Latin Hypercube Sampling. These samples, along with combinations of the maximum and minimum values of the individual distributions, were used as training data for the NIPCE model. Hemolysis was determined for these parameter combinations using the CFD approach (Equation (5)) described above. The resulting dataset of fitting parameters and corresponding hemolysis values was used to train the NIPCE model with a polynomial order of 4. This model can transform a large number of MCMC model parameter samples into a distribution of hemolysis values within seconds. More information about this reduced‐order model technique in the context of blood pumps can be found in [38] and more information as well as verification of the training process is provided in the Supporting Information (Figures S3 and S4).

Generating the training data points using CFD post‐processing on a standard laptop took approximately 60 s, and NIPCE training with these samples took 10 s. Hemolysis with uncertainty quantification was determined through 10,000 forward simulations of NIPCE for each simulation condition, taking less than a second for all six conditions simultaneously. This approach reduced the post‐processing time for 6 × 10,000 volume integrals from approximately 5 h to just over a minute, demonstrating the efficiency of the reduced‐order modeling approach.

### 2.6 Model Comparison for FDA Pump Setup

To compare the deterministic and probabilistic models, experimental hemolysis data of Conditions 1–6 from Ponnaluri et al. [35] was used. RIH values were extracted from Figure 11c of [35] using a graph digitization tool. According to Equation (4) in Ponnaluri et al. [35], the RIH values correspond to the modified hemolysis index (MIH) values, normalized to Condition 5 of the study. For the probabilistic approach, this normalization involved dividing by the median of the posterior MIH distribution of Condition 5.

## 3 Results

**FIGURE 2:**
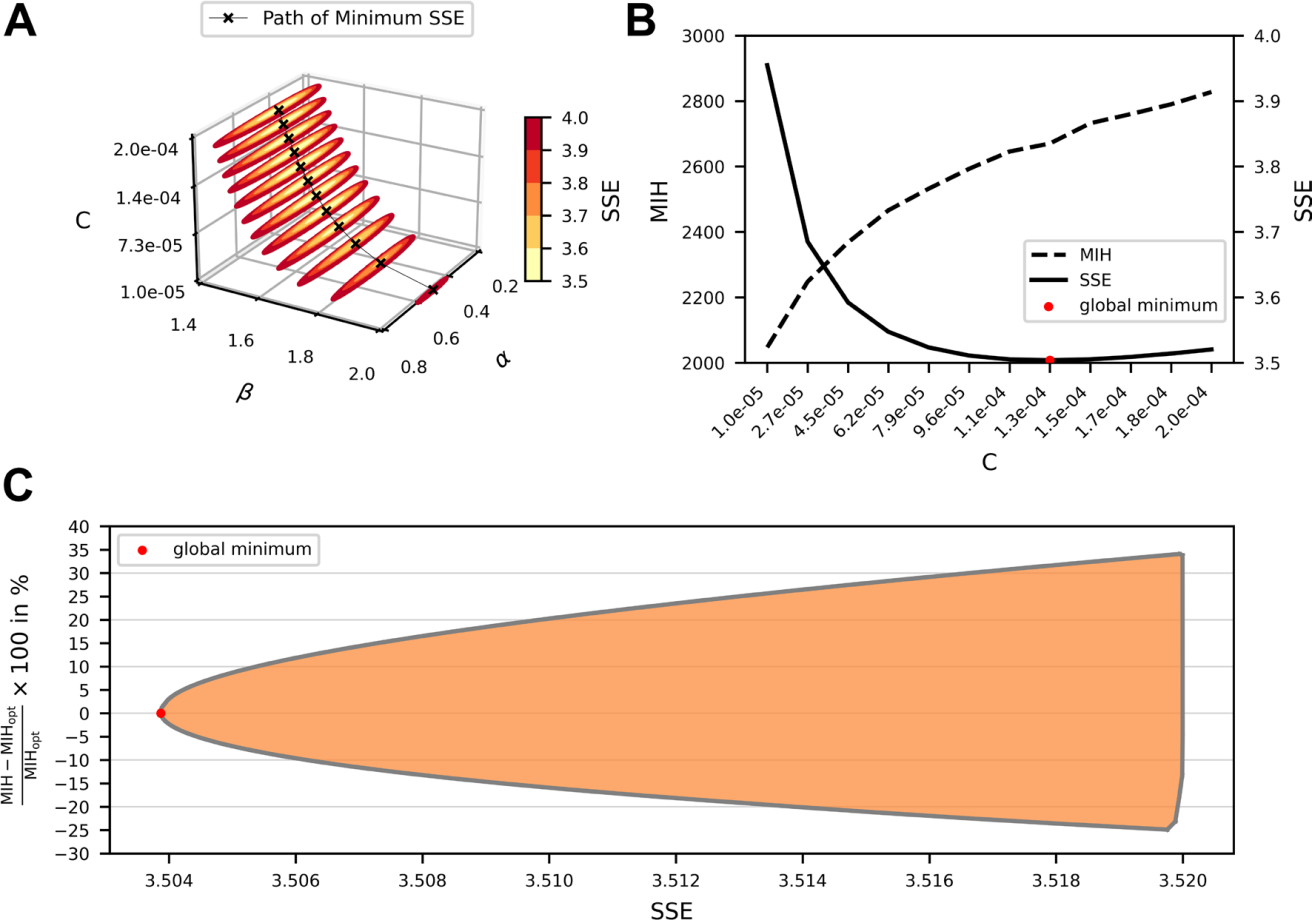
Three‐dimensional contour plots depicting the Sum of Squared Errors (SSE) of experimental data against the power law model across the parameters *C*, *α*, and *β*. The black line with cross symbols indicates the minimum SSE value for each level of *C* (panel A). In panel B the minimum SSE value for each *C* level is plotted in solid black and the corresponding Modified Index of hemolysis (MIH) for Condition 5 of the FDA blood pump is shown in dashed black. In panel (C) the range of possible hemolysis predictions for Condition 5 of the FDA pump setup is displayed for increasing SSE values. The range of possible hemolysis predictions (min, max) is shown in orange and calculated as the difference to the hemolysis value of the global SSE minimum parameter set, which is depicted by a red dot.

Figure 2A shows discrete contour slices of the Sum of Squared Errors (SSE) in a three‐dimensional plot for the parameter space *α* (0.1–1), and *β* (1.2–2.5), with 12 fixed, equidistant *C* values in the interval (1 × 10^−5^ to 1 × 10^−3^). The size of the low SSE regions decreases from high to low *C* values. To maintain a similar SSE at a given *C* level, *β* can only vary slightly (±0.02), while *α* can vary more (±0.3). This indicates that the optimal fit for HI is more sensitive to *β* than to *α*. Notably, despite different *C* levels, several regions exhibit similarly low SSE values between 3.5 and 3.6.

This is further highlighted in Figure 2B, displaying the optimal SSE value for each *C* level in solid black, along with the modified index of hemolysis (MIH) of the FDA pump setup (Condition 5) for each optimal SSE value in dashed black. The global SSE minimum is marked by a red dot. Notably, the gradient of this minimum with respect to *C* is small, resulting in a relatively flat region around the optimum. This suggests that a broader range of *C* values can yield nearly identical SSEs, indicating a degree of flexibility in fitting parameter selection. Figure 2C extends this analysis by considering the full parameter space, incorporating values around the global minimum of not only *C* but also *α* and *β*.

The plot visualizes the range of possible hemolysis predictions for Condition 5 of the FDA pump setup, demonstrating how small variations in SSE affect the prediction results. The plot shows that even a slight increase of around 0.5% in SSE from the global minimum can lead to hemolysis predictions deviating by up to 60% from the optimal value. This highlights the substantial impact of minor SSE variations on hemolysis estimations and underscores the sensitivity of the model to fitting parameter selection.

**FIGURE 3:**
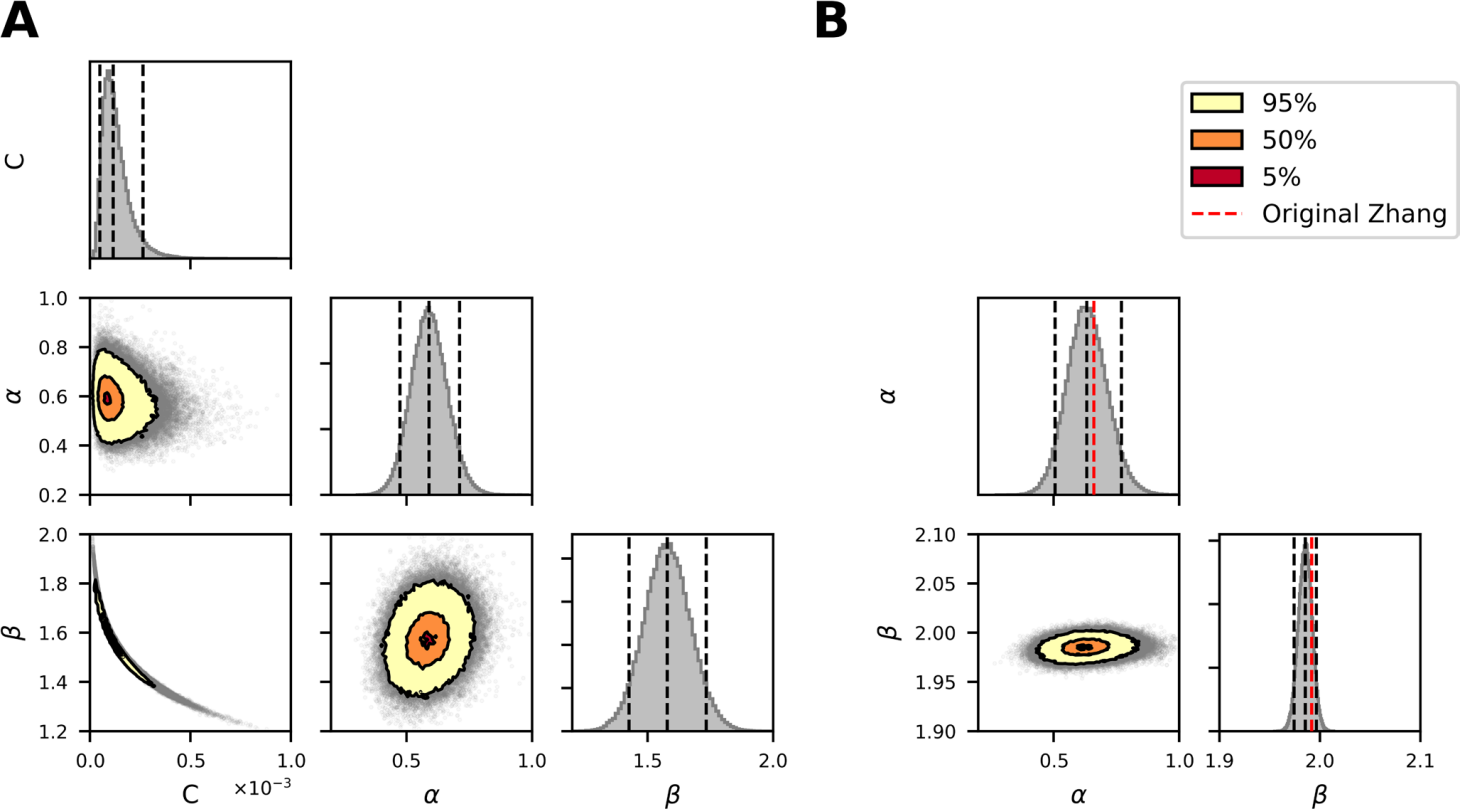
Displays the corner plot of the MCMC method for the power law model with parameters *C*, *α*, and *β* in panel (A), and for the model with fixed *C* = 1.228e‐5, with variable *α* and *β* in panel (B). The histograms show the median, along with the 5% and 95% intervals, indicated by dashed lines. The scatter plots are overlaid with contour plots representing the 95%, 50%, and 5% confidence intervals.

Figure 3A presents the results of the MCMC method in the form of a corner plot. It displays the individual distributions of the parameters *C*, *α*, and *β*, as well as their relationships in a combination of scatter and contour plots. The contour levels of the 95%, 50%, and 5% confidence intervals are depicted in yellow, orange, and red inside the scatter plots, respectively. It is clearly visible that the parameters *C* and β are correlated. This implies that no uniquely identifiable set of parameter distributions can be found, indicating redundancy in the model. An increase in one parameter can be compensated by a decrease in the other one. This observation indicates that defining *C* with a constant value and fitting the other two remaining parameters is a better strategy than optimizing all three parameters.

In Figure 3B, the corner plot with a constant *C* = 1.228e‐5, as used in the original Zhang et al. [27] model, is shown. It can be observed that through MCMC sampling, the median of the α posterior distribution has settled at 0.632, and the median of the *β* posterior distribution at 1.985. Additionally, no correlation between the parameters is evident.

**FIGURE 4:**
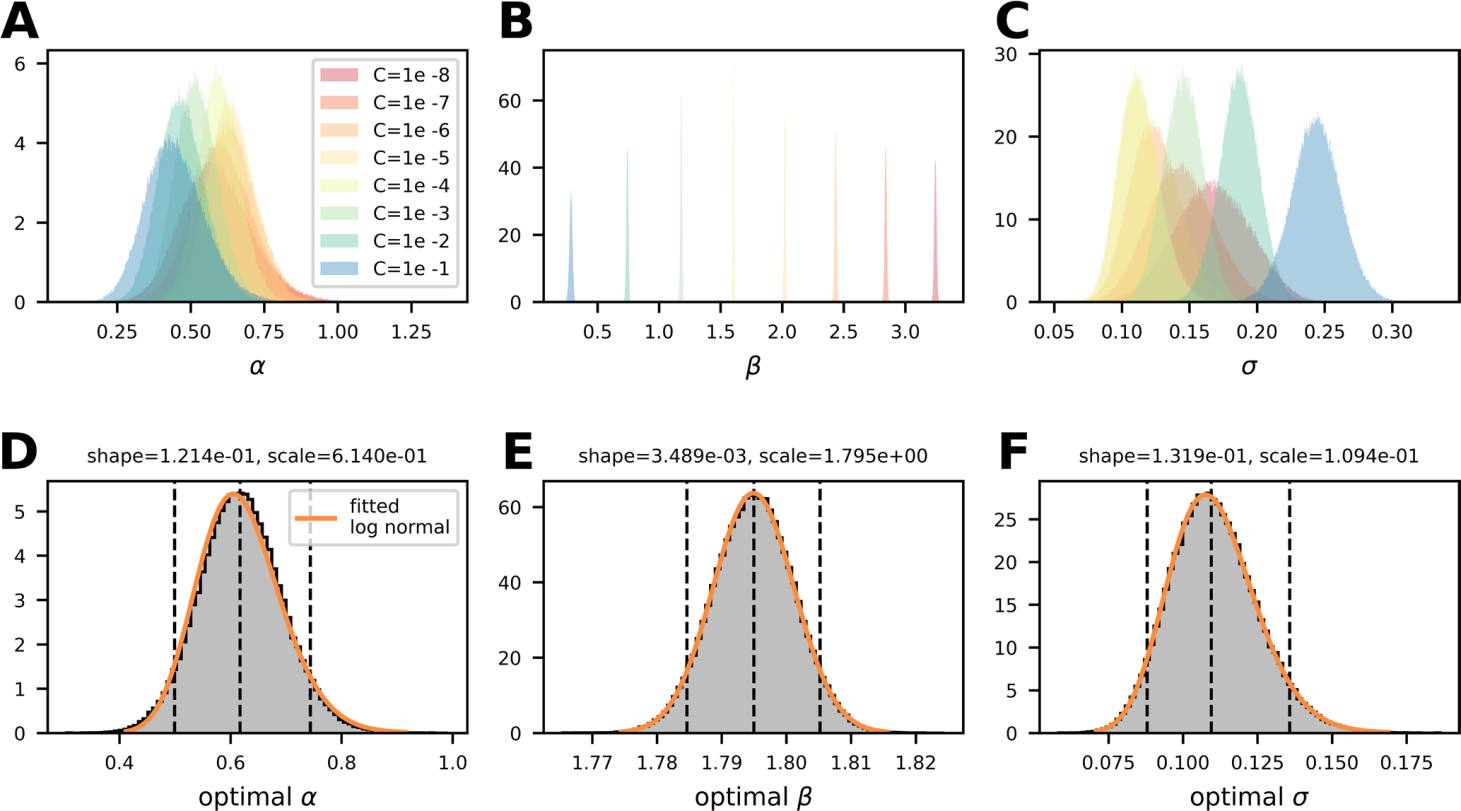
Panels (A–C) illustrate the influence of different constant *C* levels, ranging from 1 to 1e‐8, on the parameter distributions of *α*, *β*, and *σ*, respectively. Panels (D–F) depict the parameter distributions for the optimal *C* = 3.515e‐5 for *α*, *β*, and *σ*. The dashed lines indicate the 5%, 95%, and median values of the individual distributions.

However, with a constant *C* model, it is not guaranteed that the underlying experimental data can be optimally reproduced. To address this, we examined the distribution of *σ*, the parameter in the MCMC model that quantifies the residual error between the predicted and measured hemolysis data, across different fixed *C* values. By systematically varying *C*, we assessed whether the model could better represent the underlying experimental data at specific *C* values. To identify the optimal constant *C* value that minimizes *σ* and best fits the experimental data, we performed a Bayesian optimization on *σ* (Figure S5). Figure 4 shows in panels (A–C) the various distributions of *α*, *β*, and *σ* with different *C* values ranging from 1e‐8 to 1e‐1. It is evident that as the *C* value increases, the median value of the *β* distribution decreases, while the width remains relatively constant. For *σ*, which describes the differences between the observed values and the values predicted by the model, it is observed that there must be a minimal value between *C* = 1e‐8 and *C* = 1e‐1. The minimization of the median of the *σ* distribution through Bayesian optimization leads to an optimal *C* value of 3.515e‐5.

The parameter distributions of this optimal model, providing the best fit to the data and minimizing the residual error between the observed and predicted hemolysis values, are shown in Figure 4D–F. Alongside the histogram created from the MCMC samples, the 5% and 95% interval boundaries as well as the median values are indicated with dashed lines. With the given shape and scale parameters of the log‐normal distribution, as well as the constant location parameter set to 0, the depicted distributions of the optimal model can be accurately reproduced.

**FIGURE 5:**
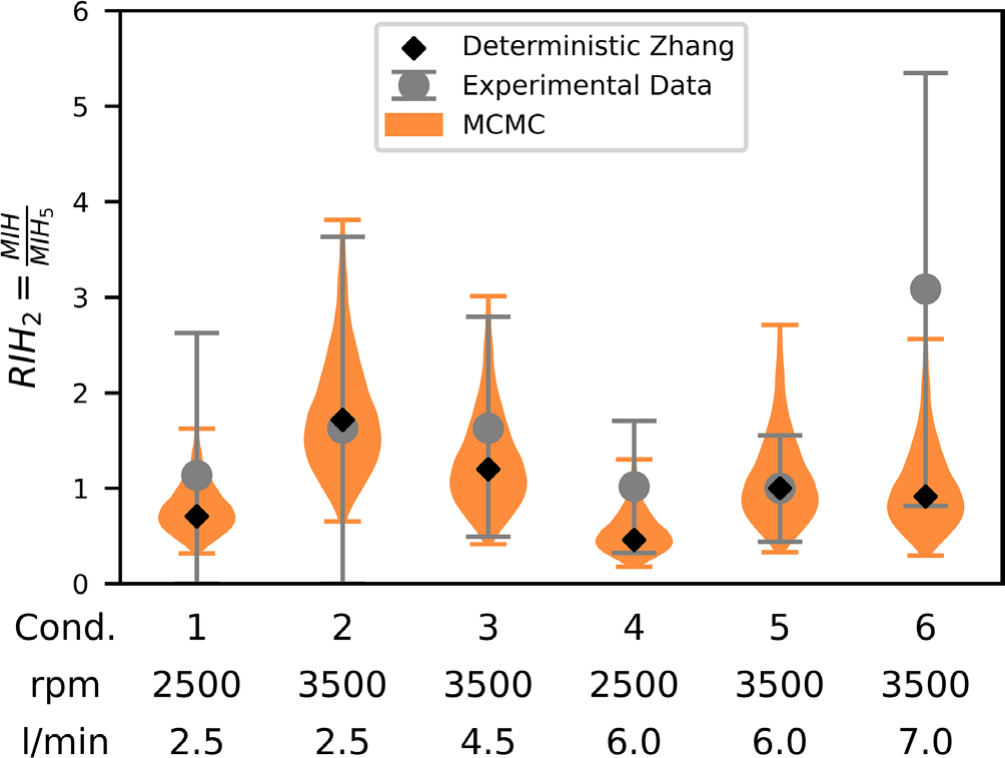
This figure compares the deterministic and probabilistic approaches with the experimental data from the FDA round robin study under six different operating conditions [35]. The deterministic model [27] is represented by black diamonds, the probabilistic model by orange violin plots, and the experimental data's mean ± SD is shown with gray error bars.

This optimal model is compared to the traditional deterministic approach and experimental data from the FDA round robin study [28] in Figure 5. The relative hemolysis index (RIH) with respect to Condition 5 is plotted for boundary conditions 1–6. The comparison between the mean values of the experimental data from Ponnaluri et al. [35] and the deterministic data of Zhang et al. [27] (black diamonds) shows that all deterministically calculated RIH values lie within the experimental measurements (mean ± SD). The new probabilistic method, represented by violin plots, shows a similar pattern regarding the median values of the distribution. In addition to the median values, the distribution of possible hemolysis values can be observed through the propagated uncertainty of the underlying experimental data. It is also evident that Conditions 1 and 4, which correspond to a rotational speed of 2500 rpm, have a smaller distribution width compared to the other conditions with a rotational speed of 3500 rpm.

## 4 Discussion

The aim of this study was to develop a universally applicable method that transfers the uncertainties from the underlying experimental data of hemolysis models into the prediction of hemolysis outcomes. Through a detailed analysis of the traditional Power Law approach, as well as the integration of reduced‐order models and Bayesian statistical methods, three main findings were identified:

1. The fitting process of uncertain experimental hemolysis data exhibits a flat global optimum, resulting in substantially different hemolysis predictions when allowing a small margin of fitting error.
2. The strong correlation between *C* and *β* prevents the MCMC approach from converging on a uniquely identifiable set of parameter distributions.
3. Considering the variance from the underlying experiments enhances the robustness of hemolysis model predictions and simplifies the comparison between experimental and simulated data.

In exploring the first finding, we employed a grid search to systematically vary the three Power Law fitting parameters (*C*, *α*, and *β*) against the measured hemolysis data, examining the sum of squared errors across all parameter combinations. The resulting SSE landscape (Figure 2A) revealed a flat global optimum for the fitted parameters. In other words, small deviations in SSE can lead to substantially different fitting parameter combinations. One reason for this flat minimum was identified to be the compensatory relationship between *C* and *β*, preserving the model output while one parameter is offset by opposite changes in the other. Importantly, this is a fundamental property of multi‐parameter power‐law models, where fitting parameters can compensate for one another based on the data they are fitted on. However, just based on the mathematical formulation of Equation (1) it is not clear that this compensatory effect in the fitting process will take place and which of the fitting parameters will contribute to this compensation. Besides the compensatory effect, the flatness of the optimum can be further amplified by three factors: the relative contribution of a fitting parameter to the model output, the range over which the associated independent variable is varied, and the inherent variance in the experimental data. Figure 2A demonstrates that the SSE is highly responsive to changes in *β* compared to *α*. Even a slight increase in *β* leads to a substantial change in the SSE, whereas similar modifications in *α* produce only minimal variations. The reason for that is that the parameter *β* exerts a strong contribution on the model output (*β* ≈ 2) and is fitted over a wide range (0–400 Pa) and is therefore more tightly constrained, leading to a steeper, more well‐defined minimum in the SSE objective function landscape. Conversely, *α*'s contribution is modest (*α* ≈ 0.6) and is fitted over a narrow range of conditions (0–1.5 s) and therefore the impact of small parameter variations on the model output becomes less noticeable. Furthermore, the variance in the underlying experimental data for each operating condition amplifies this effect because higher measurement noise means that subtle differences in the objective function are masked, allowing a broader set of parameter combinations to yield nearly equivalent SSE values. Together, these factors explain why, under the influence of contribution, range, and data variance, the objective function remains flat over a wide region of the parameter space.

A key implication of this flat SSE minimum is that even a small deviation from the global optimal SSE can lead to substantially different hemolysis predictions for the same device and operating condition. As illustrated in Figure 2B,C, hemolysis outcomes may vary by up to 60% when the SSE is allowed to increase from 3.50 to merely 3.52 (a narrow 0.5% margin). This large variation highlights the importance of quantifying parameter uncertainty because relying solely on a best‐fit parameter set can mask the possible range of plausible outcomes. Instead, acknowledging and reporting the range of solutions is essential for accurately assessing hemolysis risk and comparing experimental with simulated data. Craven et al. [26] have previously found that there are infinitely many different parameter combinations for a single operating point that lead to the same hemolysis value. This study extends that analysis, demonstrating that the proposed Power Law function might be an unsuitable choice for finding a robust representation of Couette shearing device experimental data with variance over a wider range of operating points. While there are other options that could potentially resolve this flatness issue, such as logistic or additive forms or imposing mechanistic constraints on parameters to reduce the likelihood of degenerate fits, we chose to apply a Bayesian MCMC method to incorporate uncertainties from the underlying experimental data of hemolysis models into the prediction of hemolysis outcomes.

By integrating the experimental variance directly into the parameter fitting process, we aimed not only to address the flatness of the objective function but also to demonstrate how uncertainty propagates through the model. To the best of our knowledge, this is the first study to integrate the variance from the underlying hemolysis experiments into the model parameter estimation. However, MCMC sampling revealed a persistent and strong correlation between parameters *C* and *β*, mirroring the same pattern observed in our SSE analysis (see Figures 2 and 3). This correlation leads to a nearly flat objective function landscape where a wide range of parameter combinations yield similar fits, thereby preventing convergence of the MCMC method to a uniquely identifiable set of parameter distributions. To address this challenge, we reduced the dimensionality of the multi‐parameter power‐law model by fixing *C* and sampling only *α*, *β*, and *σ*. In examining several fixed values of *C*, we observed that the residual error, quantified by *σ* as the difference between the model predictions and the measured hemolysis data, reaches an optimum at a particular value of *C*. We then employed Bayesian optimization to identify the *C* value that minimizes the median of *σ*. Minimizing *σ* in this context effectively minimizes the residuals and therefore enhances the precision of the parameter estimates for *α* and *β*. Consequently, the resulting probability distributions for these parameters more accurately reflect the true variance of the experimental data, leading to a more reliable model. This approach differs substantially from previous literature [25], which randomly samples probability distributions for *α*, *β*, and *C* from parameter intervals derived from literature values of multiple models.

Finally, to demonstrate the practical applicability of our method, we applied the Bayesian hemolysis model to the benchmark FDA round robin blood pump geometry as a post‐processing step of a CFD simulation setup. The resulting numerical hemolysis predictions were compared to experimental FDA pump data across six operating conditions. This comparison further highlights the benefit of incorporating experimental variance into the model fitting process, as the probabilistic representation of hemolysis predictions aligns well with the variability observed in the experimental measurements. The MCMC approach strengthens the robustness of relative hemolysis predictions by considering potential variance factors such as inter‐ or intra‐variability of blood samples from the underlying experiments, which can also be sources of variance in the experimental investigation of MCS devices. Furthermore, the well‐known strong sensitivity of predicted hemolysis values to variations in model parameters [25, 26, 39] is directly observable in the distribution representation of our model, facilitating a more comprehensive interpretation of the model outputs.

Testing the credibility of the MCMC method using artificially generated experimental data, which strictly follow the surface defined by the original model parameters of Zhang et al. [27] as training data, demonstrated that distributions of *α* and *β* observed in this study are solely due to the variance in the underlying data (Figure S2). This demonstrates that the resulting probabilistic representation of hemolysis values as a probability distribution only results from the underlying variance of the experimental data.

In the comparison to the FDA pump data [35] it is important to note that the modal (most probable) value of the probabilistic results closely matches the deterministic solution. This outcome reflects the fact that the deterministic solution, corresponding to the global minimum of the SSE, is identified by the probabilistic approach as the most probable solution as well. However, the probabilistic framework offers a significant advantage by quantifying and propagating uncertainties from the underlying data. As a result, while the modal value aligns with the deterministic prediction, the probabilistic approach also captures the range of plausible hemolysis outcomes arising from uncertainties of the underlying experimental data. But not all operating conditions could be predicted equally well. In particular, Condition 6 proved challenging for the numerical model as both the deterministic solution and the probabilistic solution could not predict the mean of the experimental results. That Condition 6 is particularly difficult to predict is also evident in the comparison of the numerical results of the FDA round robin study, as none of the models could reliably predict this Condition 6 [35]. This suggests that certain flow phenomena (e.g., transitional flow regimes, turbulence, flow separation) may not be fully captured by current simulations or that the experimental data themselves might be influenced by unaccounted factors. In this context, it is important to note that the experimental FDA pump data may be influenced by numerous other factors (e.g., donor differences, blood handling, uncertainty in operating conditions…) that contribute to the measured variance in hemolysis values, many of which cannot be fully accounted for in a numerical model. However, variability is not limited to the experimental side. There are also sources of uncertainty within the numerical framework that were not within the scope of this study. Specifically, our approach focuses on uncertainty in the fitted parameters of the calibrated power‐law model and does not account for uncertainties related to operating conditions, geometric fidelity (including potential manufacturing deviations or wear), or numerical modeling assumptions (e.g., mesh resolution, turbulence modeling choices, and rheological models for blood). Each of these factors can influence the predictive accuracy of hemolysis models in practical blood pump simulations and contribute to overall uncertainty. Accordingly, while this study enhances the understanding of fitting parameter‐related variability, it should be considered as one component within a broader uncertainty quantification framework. Additionally, it is important to note that the raw data from Zhang et al. [27] examined bovine blood, while the FDA pump experiments [35] investigated porcine blood. Consequently, the comparison to the FDA pump data serves only as an illustrative example of how the probabilistic hemolysis model simplifies the comparison to experimental data. Nonetheless, despite the different donors, a relative comparison between simulation and experiment is still possible.

However, the problem of accurately predicting absolute hemolysis values is not solved by the probabilistic model. Absolute hemolysis values of the probabilistic approach are within the same order of magnitude as those predicted by classical power law models. This issue does not appear to be due to the variance in the underlying experimental data but, as discussed in the literature, seems to result from more fundamental modeling issues. In particular, the application of calibration data from simplified experimental setups to more complex flow environments introduces additional uncertainties, as controlled shear exposure experiments may not fully capture the range and type of stresses and range of residence times encountered in realistic pump flows. Moreover, the inability to resolve all turbulence scales and individual RBC dynamics in numerical simulations further limits predictive accuracy, as key flow structures that influence hemolysis may not be fully captured [24].

## 5 Conclusions

This study highlights fundamental mathematical limitations in the fitting process of the classic multi‐parameter power law models with uncertain experimental data and successfully demonstrates how the inherent uncertainty in hemolysis experiments can be captured and implemented into numerical blood damage models. It further shows that incorporating fitting parameter variability through MCMC substantially enhances the robustness of hemolysis model predictions. The current gold standard of relative comparisons is strengthened by incorporating the variance of the underlying experiments, providing a stronger foundation for comparing simulated hemolysis outcomes with in vivo experiments. The developed method can easily incorporate further experimental datasets encompassing various stress types, donor species, and a higher number of repetitions. Due to the Bayesian nature of our method, future work could include more data points from different experiments with varied shear conditions and exposure times by updating the posterior distribution with new datasets. This approach has the potential to result in a comprehensive model that incorporates all the experimental knowledge of the scientific community, setting a new standard of predictive accuracy in hemolysis modeling.

## Author Contributions

All authors contributed to the study conception and design. C.B. developed the numerical model, performed the simulations, gathered, analyzed and discussed the results. M.N. and U.S. were involved in the analysis and discussion of the results. M.N. supervised the project. C.B. wrote the manuscript based on the input of all co‐authors. All co‐authors read and approved the final version of the manuscript.

## Ethics Statement

The authors have nothing to report.

## Consent

The authors have nothing to report.

## Conflicts of Interest

The authors declare no conflicts of interest.

## Supporting information


**Data S1.** Supporting Information.

## Data Availability

The data that support the findings of this study are available in https://zenodo.org/records/12820264 and Supporting Information. Further data are available upon reasonable request.
