# Supplementary material for: Toward Uncertainty‐Aware Hemolysis Modeling: A Universal Approach to Address Experimental Variance
Source: Int J Numer Method Biomed Eng. 2025 May 14;41(5):e70040. doi: 10.1002/cnm.70040 (PMC12076269; doi:10.1002/cnm.70040)
Supplement: Supplementary file 1 — Data S1. Supporting Information. [file CNM-41-e70040-s001.docx]

**Towards Uncertainty-Aware Modeling: A Universal Approach to Address Experimental Variance**

Christopher Blum^a,*^, Ulrich Steinseifer^a^, Michael Neidlin^a^

1. Department of Cardiovascular Engineering, Institute of Applied Medical Engineering, Medical Faculty, RWTH Aachen University, Aachen, Germany

#

# *Correspondence:

Name: Christopher Blum

Address: Institute of Applied Medical Engineering, Pauwelsstraße 20

52074 Aachen, Germany

Email address: [blum@ame.rwth-aachen.de](mailto:blum@ame.rwth-aachen.de)

**Supplementary Information**

This document offers a detailed explanation of the methodologies applied in our research. The novel probabilistic approach is based on the Markov Chain Monte Carlo (MCMC) method and a reduced order model method called non-intrusive polynomial chaos expansion (NIPCE). Both methods as well as a more detailed description of the optimization method used in the main manuscript will be presented in the following.

**MCMC**

In this section, we present the trace plots for the four MCMC chains used in the analysis (Figure S1). These plots help to visualize the convergence and mixing of the chains. Each chain was run with 50,000 samples, following a burn-in period of 1,000 samples.


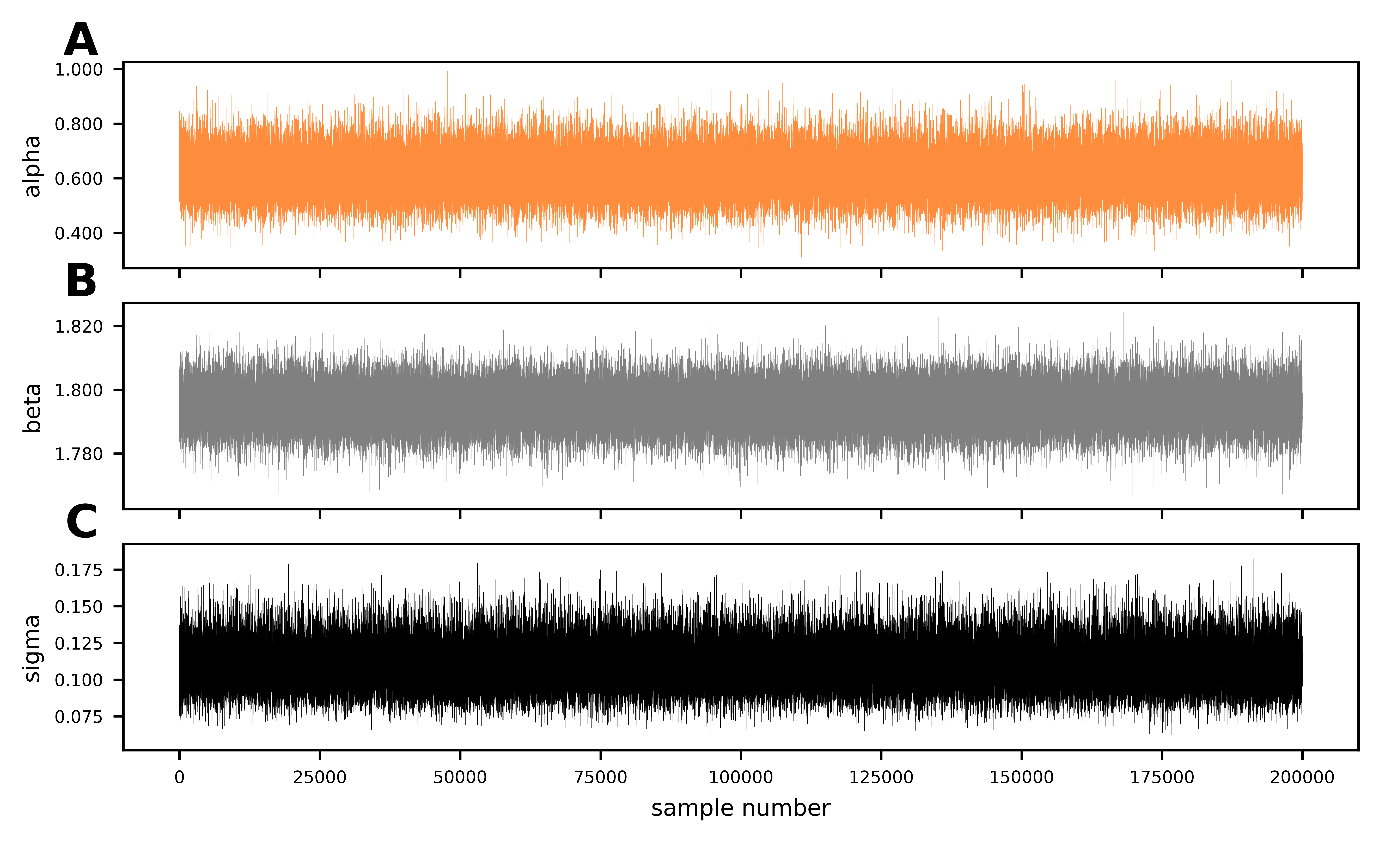


Figure S1:Trace plots of the parameters α, β and σ in A, B and C respectively.

All traces appear to be stable with no visible trends, indicating good stationarity. The samples cover the entire range without sticking to specific values. To further assess the convergence of the MCMC chains, we calculated the Gelman-Rubin Diagnostic (R-hat) for each parameter to be 1. An R-hat value close to 1 indicates good convergence.

To illustrate the impact of experimental variance on the results of the MCMC method, synthetic experimental data without variance were generated over the same interval as the experimental data. These synthetic data are positioned exactly on the surface defined by Zhang's parameter values. As shown in Figure S2 A, the MCMC method yields Zhang's parameter values precisely, without any distribution and σ being 0, when using the synthetic data. This demonstrates that the distributions of the model parameters resulting from the MCMC method and the actual experimental data directly originate from the variance of the experimental data (Figure S2 B).

**
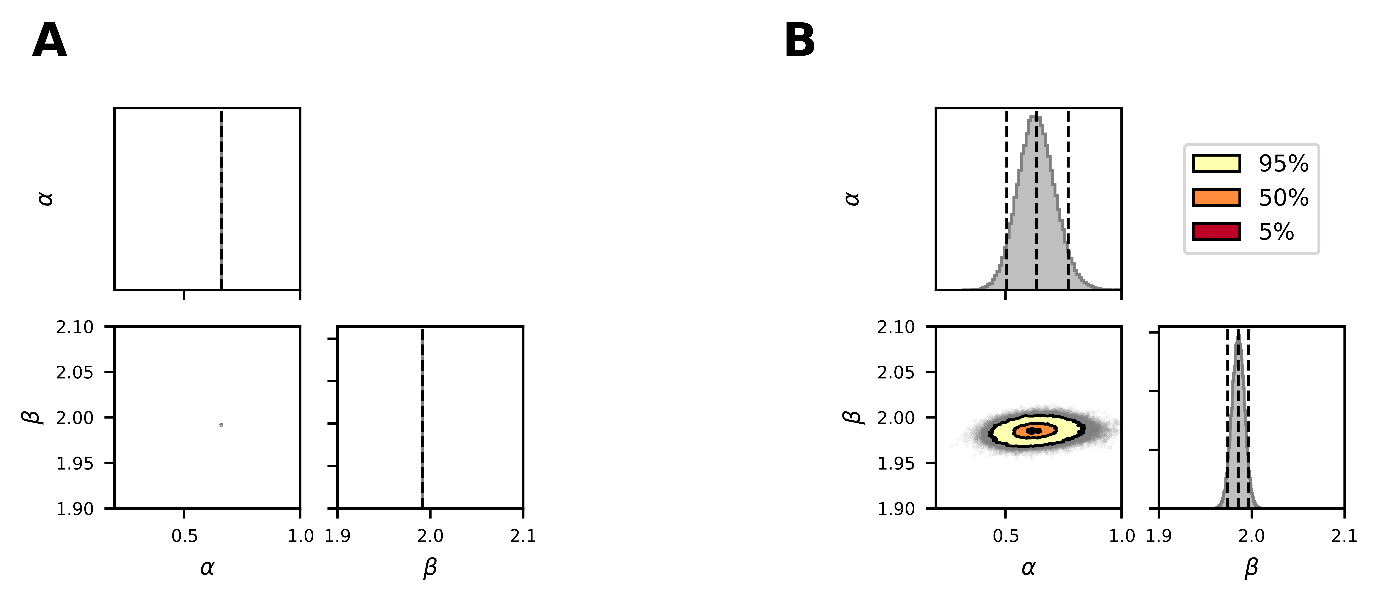
**

Figure S2: Corner Plot comparison of MCMC method with constant C=1.228e-5 and synthetic experimental data without variance (A) and with the actual experimental data including variance (B).

**NIPCE**

The posterior distributions of the model parameters (C, α, β, and σ) obtained from the MCMC sampling were first fitted using log-normal probability distributions.
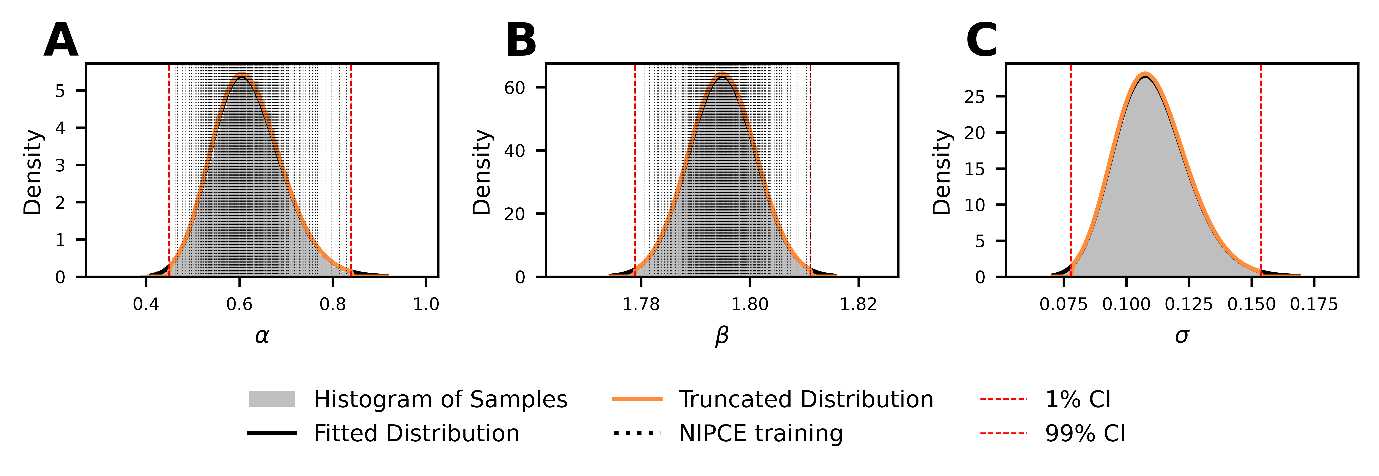


Figure S3: Posterior Distributions of the model parameters α, β and σ are shown in Panels A, B and C respectively. Fitted log-Normal distributions are indicated by black lines and truncated distributions by orange lines. The 1% and 99% truncation criterions are indicated by dashed red lines. For α and beta the Latin Hypercube training points are shown with black dotted lines.

To ensure robustness and avoid the influence of extreme values, these distributions were truncated at the lower and upper ends of the 99% confidence intervals (Figure S3). The individual fitted distributions were then combined to form a multivariate distribution.

From this multivariate distribution, 200 samples were drawn using Latin Hypercube Sampling (LHS), a method that ensures a well-distributed sampling across the parameter space defined by the 99% confidence intervals. Additionally, to include potential boundary effects, combinations of the maximum and minimum values of the individual distributions were added, resulting in a total of 209 (for the C, α, β models) or 204 (for the α, β models) training points. Each of these parameter sets was used to compute hemolysis values via the CFD post-processing approach described in the main text, thus providing the data needed to train the NIPCE model.

A fourth-order polynomial expansion was selected for the NIPCE model, balancing complexity and computational efficiency. Once trained, the NIPCE model enables rapid transformation of large numbers of MCMC samples into corresponding hemolysis value distributions within seconds.

The accuracy and reliability of the NIPCE model were verified by comparing its predictions to the original CFD results. Although this procedure was conducted for all conditions of the FDA pump setup, Figure S4 illustrates a representative example for Condition 5.


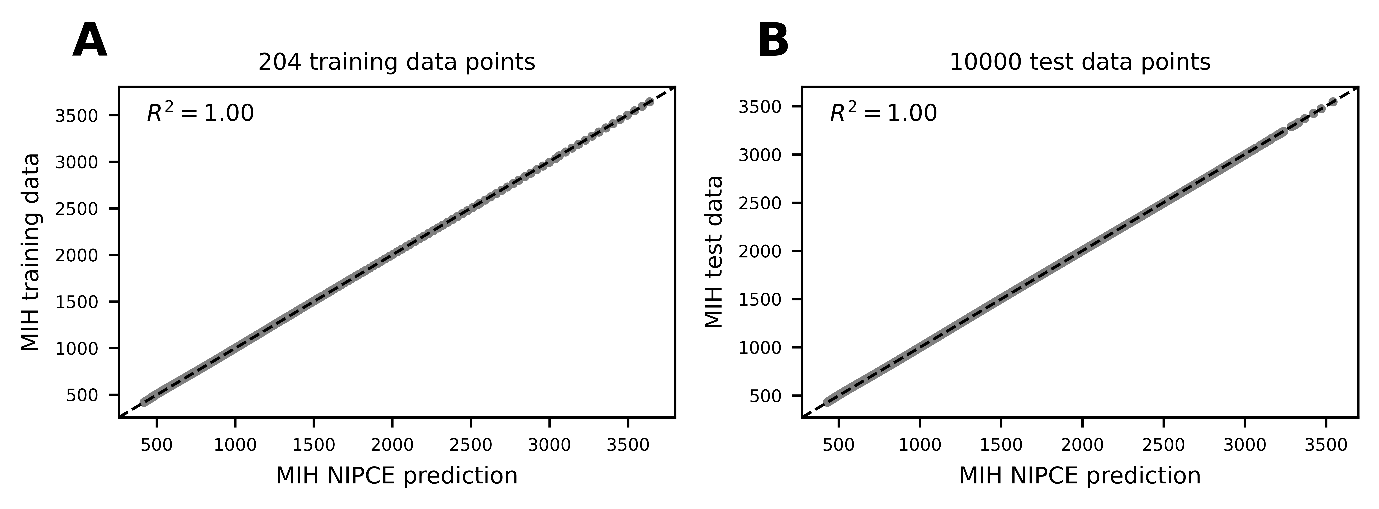


Figure S4: Correlation plot of NIPCE prediction and CFD value for MIH for training data points (A) and 10000 test data points for Condition 5 of the FDA pump.

In panel (A), the NIPCE model reproduces hemolysis values accurately for the training data, while in panel (B), it demonstrates robust predictive capability for an additional 10,000 randomly sampled parameter combinations within the 99% confidence intervals. These results confirm that the NIPCE method can reliably predict hemolysis values within the entire training space defined by the posterior distributions.

**Parameter optimization**

Bayesian optimization is an iterative process that uses a surrogate model to approximate the objective function and an acquisition function to decide where to sample next. In our study, this method was applied to find the optimal C value by minimizing the median of the σ distribution. The optimization process used a Gaussian Process (GP) as the surrogate model to approximate the relationship between C values and the median of the σ distribution. Additionally, an Acquisition Function guided the selection of the next C value to evaluate. The Bayesian optimization was performed on the parameter σ, with C values ranging from 1e-6 -1e-3. For each C value, the posterior distributions of α, β and σ were evaluated. As can be seen from Figure S5 the optimization process identified 3.515e-5 as the optimal C value after 100 Iterations.


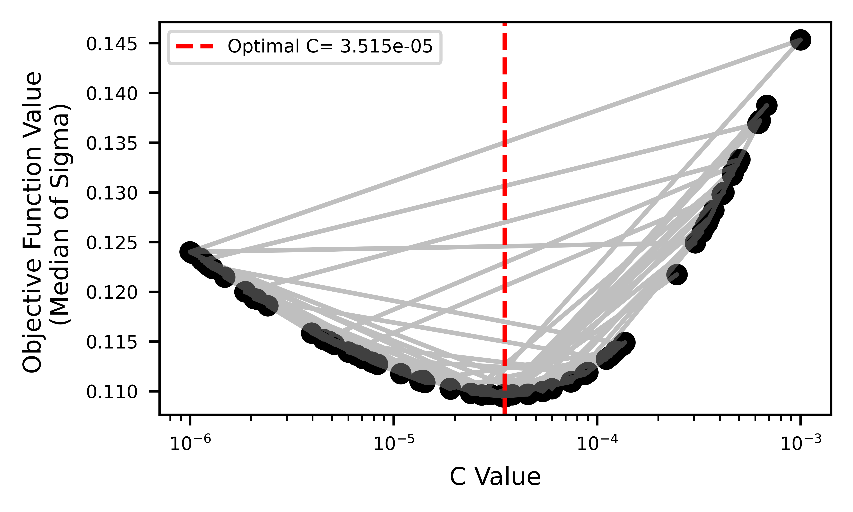


Figure S5: Optimization history of 100 iterations finding the smallest median of σ = 3.515e-5 as indicated by a red vertical line. Black points indicate individual objective function realizations and grey lines show the how the algorithm advanced from one point to another.
